# Machine learning-based evaluation of risk factors for carbapenem-resistant *Klebsiella pneumoniae* dissemination in neonatal units

**DOI:** 10.1128/msystems.00909-25

**Published:** 2025-09-15

**Authors:** Xiao Liu, Muxiu Jiang, Jinzhi Zhang, Heng Li, Yina Liu, Jiaqi Zhang, Xia Chen, Jun Bu, Shichang Xie, Menghan Zhang, Ning Dong, Qing Cao, Zhemin Zhou

**Affiliations:** 1 Key Laboratory of Alkene-carbon Fibres-based Technology & Application for Detection of Major Infectious Diseases, MOE Key Laboratory of Geriatric Diseases and Immunology, Cancer Institute, Suzhou Medical College, Soochow University, Suzhou, China; 2 National Key Laboratory of Intelligent Tracking and Forecasting for Infectious Diseases, National Institute for Communicable Disease Control and Prevention, Chinese Center for Disease Control and Prevention 12415, Beijing, China; 3 Department of Infectious Disease, Shanghai Children’s Medical Center, School of Medicine, Shanghai Jiao Tong University 12474, Shanghai, China; 4 Emergency and Critical Care Center, Intensive Care Unit, Zhejiang Provincial People's Hospital (Affiliated People's Hospital, Hangzhou Medical College) 74678, Hangzhou, Zhejiang, China; 5 Jiangsu Province Engineering Research Center of Precision Diagnostics and Therapeutics Development, Soochow University 63297, Suzhou, China; 6 Department of Neonatology, Shanghai Children’s Medical Center, School of Medicine, Shanghai Jiao Tong University 12474, Shanghai, China; 7 Iotabiome Biotechnology Inc., Suzhou, China; 8 Suzhou Center for Disease Control and Prevention, Suzhou, China; 9 School of Public Health, Zhejiang University School of Medicine 26441, Hangzhou, China; Drexel University, Philadelphia, Pennsylvania, USA

**Keywords:** neonates, *bla*_NDM-1_, multi-drug resistance plasmid, phylogeny

## Abstract

Healthcare-associated infections (HAIs), particularly in neonatal intensive care units (NICUs), pose significant challenges due to neonates’ vulnerability and the rapid infection spread. However, risk factors facilitating pathogen persistence and dissemination have not been comprehensively investigated. This study aims to track HAI transmission pathways in NICUs and identify key risk factors contributing to the persistence and spread of carbapenem-resistant *Klebsiella pneumoniae* (CRKP). We analyzed CRKP epidemiology and population dynamics in neonatal patients at a pediatric hospital in China over 8 years. Random forest models identified primary risk factors for CRKP persistence and outbreaks, focusing on clonal spread, healthcare groups (HGs), and plasmid dynamics. Three major clonal outbreaks involving ST14 and ST433 strains were identified, highlighting the critical role of clonal dissemination in NICUs. Complex transmission patterns, characterized by periods of dormancy and resurgence, suggest the existence of underlying reservoirs. HGs were implicated in the short-term transmission of CRKP, with >80% of infection clusters involving patients from the same HG. Plasmids emerged as critical factors in the long-term persistence of CRKP, with shifts in plasmid prevalence corresponding to outbreak periods. This study advances our understanding of CRKP transmission dynamics in NICUs, highlighting the multifaceted roles of clonal dissemination, HGs, and plasmid-mediated persistence. Our findings emphasize the need for enhanced infection control measures targeting both intra- and inter-group transmissions and plasmid surveillance.

**IMPORTANCE:**

This study provides a detailed analysis of carbapenem-resistant *Klebsiella pneumoniae* (CRKP) transmission dynamics in neonatal intensive care units (NICUs) over eight years, utilizing 64 isolates and applying machine learning to identify risk factors associated with persistence and spread. Through phylogenetic analyses, we uncovered three clonal outbreaks and linked healthcare group (HG) interactions, bacterial genotypes, and plasmid prevalence to short- and long-term CRKP transmission. We identified that HGs are primary mediators of rapid, short-term transmission, while specific plasmids play an extended role in maintaining CRKP presence across multiple patient cohorts and bacterial strains. This finding suggests the existence of latent reservoirs or periodic reintroductions from external sources, thus reshaping the understanding of NICU-associated pathogen transmission and persistence.

## INTRODUCTION

Infection outbreaks in intensive care units (ICUs) pose significant concerns due to the heightened vulnerability of patients and the potential for rapid transmission within these settings (1). Healthcare-associated infections (HAIs) involving multidrug-resistant (MDR) pathogens affect ~30% of ICU patients (2), contributing to increased healthcare costs and adverse patient outcomes (3, 4). Neonatal ICUs (NICUs), which care for critically ill neonates with immature immune systems, are particularly susceptible to infection outbreaks (5). The growing threat of antimicrobial resistance (AMR) among pathogens, coupled with limited therapeutic options for neonates (6), highlights the urgent need to elucidate the patterns of infection in NICUs to facilitate early detection and break transmission chains.

Despite the controlled ICU environment, complex transmission pathways exist, complicating outbreak investigations (7). Infectious outbreaks have been linked to contaminated surfaces, medical devices, water tanks, and healthcare workers’ clothing or hands (8). However, the lack of long-term surveillance hinders comprehensive evaluation of factors contributing to the introduction and spread of bacteria in ICUs. Notably, while bacterial clonal spread is well recognized as a major driver of nosocomial outbreaks, the role of genetic elements, including plasmids, in facilitating long-term persistence and inter-strain transmission of CRKP, particularly in NICU settings, has not been systematically investigated (9).

*Klebsiella pneumoniae* is a leading cause of neonatal infections, accounting for over 20% of fatalities in bloodstream infections (10). The emergence of carbapenem-resistant *K. pneumoniae* (CRKP), particularly those producing the New Delhi metallo-β-lactamase (NDM) encoded by the *bla*_NDM_ genes, has further complicated treatment efforts (11). These *bla*_NDM_ genes are often carried on plasmids, facilitating both local transmission and inter-species dissemination (11). In China, the prevalence of *bla*_NDM_ has been steadily increasing, particularly in pediatric patients, contributing to more frequent NICU outbreaks (12).

Predicting CRKP outbreaks poses significant challenges due to the multifaceted nature of transmission and the genetic adaptability of the pathogen (13). Machine learning-based approaches, such as random forest models, offer promising avenues for identifying risk factors and predicting CRKP spread by analyzing multi-dimensional data, including patient histories, environmental factors, and genomic data (14). These tools can enhance outbreak preparedness by pinpointing critical variables associated with pathogen dissemination, enabling earlier interventions. In this study, we analyzed 64 CRKP isolates collected from neonatal patients over 8 years, delineating transmission patterns during infection outbreaks. We employed random forest models to evaluate risk factors contributing to the dissemination of CRKP isolates in neonatal units, offering new insights into how plasmid-mediated gene transfer and the healthcare environment sustain pathogen reservoirs and contribute to the recurrence of outbreaks.

## MATERIALS AND METHODS

### Clinical data collection and study design

A total of 65 CRKP isolates were collected from 59 neonates admitted to the Shanghai Children’s Medical Center (SCMC) from 2013 to 2020. We retrospectively collected clinical data for each patient, including age, sex, ward, birth history, attending healthcare group (HG), underlying diseases, invasive procedures, and clinical outcomes. The study’s inclusion criteria are as follows: (i) infants aged ≤28 days; (ii) infants with infection symptoms and positive culture for *K. pneumoniae* in clinical samples; (iii) infants with complete clinical records; (iv) *K. pneumoniae* isolates that exhibited resistance to one or more carbapenem antibiotics, such as imipenem, meropenem, and ertapenem; (v) only one isolate was kept for each sample taken from the patients. *K. pneumoniae* isolates that were obtained from the same patient at different time points were considered different, to preserve potential genetic variations during treatment.

### Antimicrobial susceptibility testing

The susceptibility of *K. pneumoniae* isolates to carbapenem antibiotics was validated using the disk diffusion assay of meropenem and imipenem (Oxoid), following the Clinical and Laboratory Standards Institute guidelines (2022). Carbapenem resistance was defined as *K. pneumoniae* with an inhibition zone diameter not exceeding 19 mm. *Escherichia coli* ATCC 25922 served as a quality control.

### Whole-genome sequencing

The silica gel columns (D3146, HiPure Bacterial DNA kit) were used for DNA purification and recovery of CRKP isolates. Genomic libraries were prepared according to Illumina’s standard genomic DNA library preparation procedure (VAHTS Universal DNA Library Prep kit for Illumina V3) based on the manufacturer’s instructions. Sequencing was performed on an Illumina NovaSeq 6000 using the S4 reagent kits (v.1.5), generating paired-end reads with a read length of 2 × 150 bp reads.

Additionally, eight isolates were selected for sequencing on a GridION device (Oxford Nanopore Technologies, Oxford, UK) following the manufacturer’s procedures. Libraries were prepared with the Ligation Sequencing Kit (SQK-NBD114.24) following the manufacturer’s protocol and sequenced using R10.4.1 MinION Flow Cells (FLO-MIN114).

### Bioinformatics

FastQC (v.0.12.1) was used to evaluate the quality of Illumina reads. No isolates were excluded due to poor sequencing quality. The sequencing reads of each isolate were quality-trimmed using the “prepare” module in EToKi (15). Cleaned reads were assembled into contigs using the “assemble” module in EToKi, which internally calls SPAdes (v.3.15.2) for short-read assembly with default parameters.

NanoPlot (v.1.44.1) was used to evaluate the quality of Nanopore reads. Then, the “assemble” module of EToKi was also employed to do hybrid assembly of both Nanopore and Illumina reads for the eight isolates with Nanopore reads. In this process, Flye (v.2.8.3) was used for initial Nanopore read assembly, while SPAdes (v.3.15.2) was applied for hybrid assembly using both long and short reads. The assemblies generated by both strategies were then compared based on their N50 values. One isolate (HB021691) was excluded from downstream comparative analyses due to discrepancies between its Illumina and Nanopore sequencing results, which suggested potential sample contamination. To ensure the accuracy and consistency of the genomic data, only isolates with concordant results from both sequencing platforms were retained for further analysis. All assembled genomes were annotated using Prokka (16). The sequence types (STs), antimicrobial resistance genes ( ARGs), and virulence determinants were predicted using Kleborate (17), and the plasmids were predicted using KleTy (11). Comparisons of the plasmids were conducted using BLAST Ring Image Generator.

### Phylogenetic analysis

The maximum-likelihood (ML) tree of 64 isolates was calculated using the “align” and “phylo” modules in the EToKi package (15). Briefly, EToKi employs minimap2 to align all genomes onto a reference sequence (HB021614) to obtain a multi-sequence alignment and estimate an ML tree based on the alignment using IQ-TREE (18). Furthermore, we estimated one ML tree for each infection outbreak in the NICU after aligning associated genomes to the corresponding complete genomes we obtained (ST14: HB021614, ST433: HB021607). The recombinant regions were removed using RecHMM (19). Additionally, we compared the genomes of ST14 and ST433 with additional publicly available *K. pneumoniae* genomes deposited in EnteroBase and GenBank. All resulting trees were visualized online using iTOL v.6 (20).

### Random forest models

The contribution of risk factors to CRKP dissemination was evaluated using two random forest models based on the scikit-learn library (21). The first model aimed to identify the key factors contributing to the persistence of CRKP isolates over time, using genetic data (bacterial genotypes, plasmids) and clinical metadata (healthcare groups, wards) as independent variables, and the time differences between isolates as dependent variables. A RandomForestRegressor model was used, and the hyperparameters (n_estimators, max_features, and max_depth) were optimized using the GridSearchCV function, with fivefold cross-validations to minimize overfitting. The final model used 100 trees with a maximum depth of 12, with a mean squared error of 0.86. The feature importance was evaluated using the permutation_importance function (22). The second is a RandomForestClassifier model designed to predict outbreak-associated genotypes (ST433, ST14-C1, and ST14-C2) based on the presence/absence of accessory genes. A similar data split was used, with cross-validation ensuring robustness. Hyperparameters were also optimized using grid search, and the feature importance was evaluated using permutation importance.

### Statistical analysis

The significance of each risk factor contributing to the transmissions was measured by its uneven distribution along the timeline. To this end, the clinical metadata and strain genotypes associated with all patients were permuted 1,000 times, and the frequencies of observing multiple patients with the same metadata in a certain time period (1, 2, 3, …, 12 months) were calculated and compared to the actual frequencies. Given the average frequencies of observing repetitive metadata in a certain period as $\hat{\mu }$, with a standard deviation of $\hat{\sigma }$, the actual frequencies, *f*, were considered significant if


$$f-\hat{\mu }\geq 3.8\hat{\sigma }$$

with an expected probability of <0.01, after the Bonferroni adjustment.

## RESULTS

### Clinical features of neonates and CRKP isolates

We retrospectively studied 65 CRKP isolates from 59 neonates at a pediatric teaching hospital from January 2013 to December 2020 (Table S1). One isolate was excluded from downstream analyses due to inconsistency between its Illumina and Nanopore sequencing results, resulting in a final data set of 64 CRKP isolates from 58 neonates (Table S2). Of the neonates, 53.4% (31/58) were premature, with a median age of 4.11 days. Admissions included 56.9% (33/58) to the NICU, 12.1% (7/58) to the cardiac intensive care unit (CICU), and 31.0% (18/58) to the neonatal unit (Table 1). Healthcare was provided by 21 distinct HGs labeled A–N, each with separate attending physicians and designated beds. Less than half (9/21) of the HGs were associated with multiple enrolled patients, with groups D (11 patients), A (10 patients), and C (8 patients) leading the care. The NICU and neonatal unit shared many HGs, whereas CICU HGs were unique.

**TABLE 1:** Clinical characteristics of carbapenem-resistant *Klebsiella pneumoniae* isolates in neonates

Parameter	Result for carbapenemase-encoding plasmids and sequence types						Summary
	pSCMC1			pSCMC3		Others	
	ST14	ST433	Other STs	ST14	Other STs	Other STs	
Median age (day)	1.27	2.47	11.83	4.73	20.29	12.42	7.38
Gestational age							
Full-term	2	2	8	5	3	9	29
Preterm	9	5	5	14	0	2	35
Gender							
Female	0	2	6	9	2	3	22
Male	11	5	7	10	1	8	42
Ward							
Neonatal unit	0	1	7	3	2	5	18
NICU	11	6	2	16	0	4	39
CICU	0	0	4	0	1	2	7
Clinical intervention							
Incubator	8	3	4	12	0	0	27
Pre-infection phototherapy	8	4	3	13	1	1	30
Mechanical ventilation	5	2	5	7	1	6	26
Intravenous catheterization	5	3	6	12	1	4	31
Urethral catheterization	0	2	4	3	1	3	13
Prior surgery^*a*^	2	2	6	6	1	4	21
Underlying disease^*b*^							
CHD	0	0	6	1	1	7	15
CIM	4	0	1	2	0	1	8
NEC	2	3	3	5	0	0	13
NRDS or pneumonia	5	0	3	10	0	1	19
Others	0	4	0	1	2	2	9
Clinical outcome							
Survival	9	7	12	17	3	8	56
Death	2	0	1	2	0	3	8

^*a*^ See Table S1 for details.

^*b*^ CHD, congenital heart disease; CIM, congenital intestinal malformations; NEC, necrotizing enterocolitis; NRDS, neonatal respiratory distress syndrome; UTI, urinary tract infections; Others, intrauterine infection, sacrococcygeal tumors, liver failure, purulent meningitis, neonatal hyperbilirubinemia, or scalp hematoma.

Among the 58 neonates, 25.9% (15/58) had congenital heart disease, with nine having undergone congenital surgery before infection. Necrotizing enterocolitis occurred in 19% (11/58) of patients, most being preterm infants. Neonatal respiratory distress syndrome and neonatal pneumonia (not caused by CRKP) were observed in 19% (11/58) and 12.1% (7/58) of patients, respectively (Table 1; Table S1). Additionally, 10.3% (6/58) had congenital intestinal malformations, with five requiring intestinal surgery. The average hospital stay was 35 days (range 1–73 days), with 87.9% (51/58) of patients either cured or showing clinical improvement.

### Genotypes of CRKP isolates

**Fig 1:**
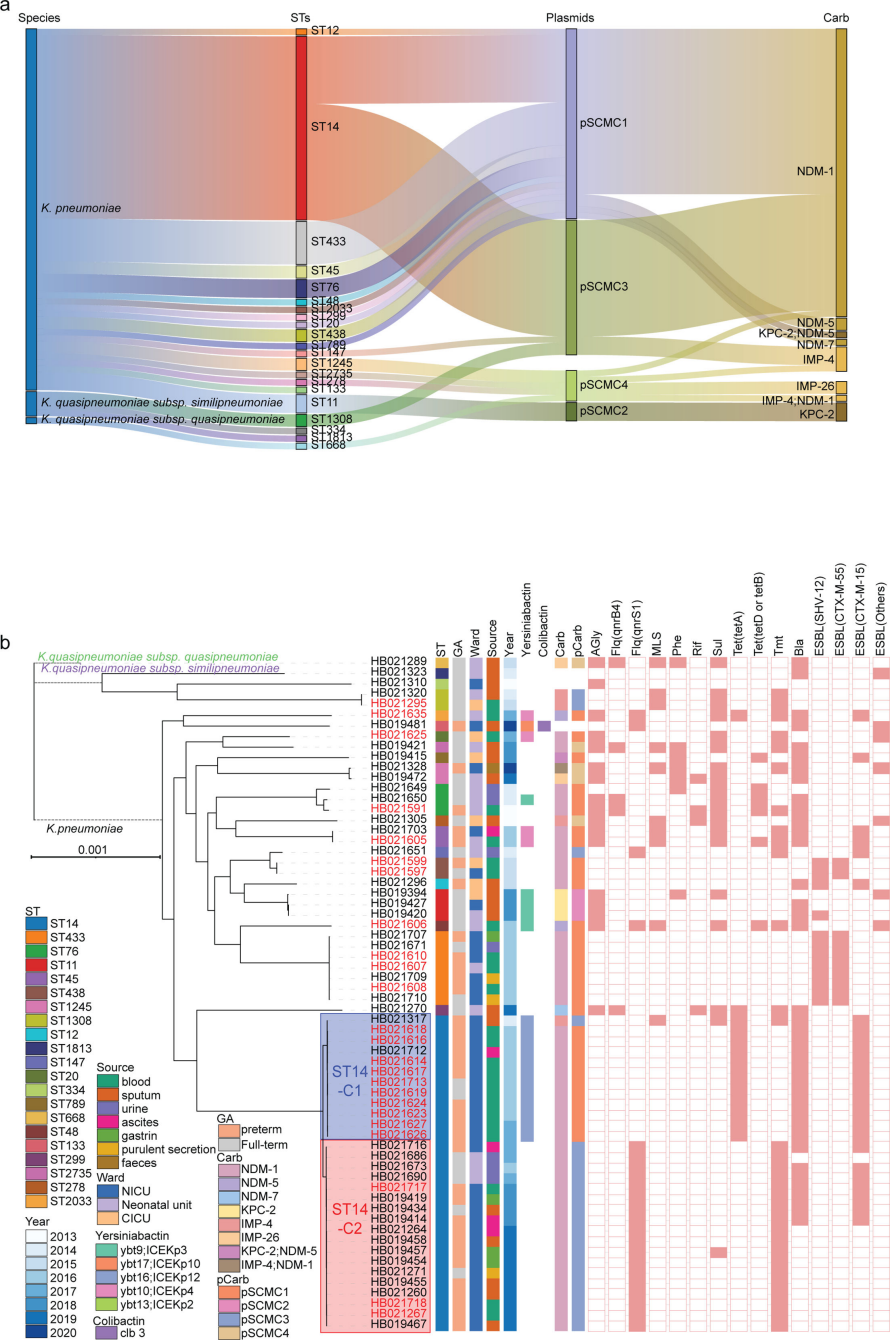
Genotypes of carbapenem-resistant *Klebsiella pneumoniae* in neonates. (**a**) A Sankey diagram of the relationships among the species, STs, plasmids, and carbapenem-resistance genes (from left to right). (**b**) The maximum-likelihood phylogeny of the 64 isolates. Branches between different subspecies were shortened for better visualization. Isolates from the bloodstream were labeled in red. The colored boxes show the three clones associated with NICU outbreaks. The metadata is shown on the right-hand side. GA, gestational age; Carb, carbapenem-resistance genes; pCarb, carbapenemase-encoding plasmids; AGly, aminoglycosides; Flq, fluoroquinolones; MLS, macrolides; Phe, phenicols; Rif, rifampin; Sul, sulfonamides; Tet, tetracyclines; Tmt, trimethoprim; Bla, beta-lactamases; ESBL, extended-spectrum beta-lactamases.

We obtained and genomically sequenced 64 CRKP isolates from various sources, primarily from blood (37.5%), sputum (31.3%), and urine (10.9%). Illumina sequencing generated high-quality paired-end reads, with Q30 values exceeding 93%, an average GC content of 57%, and mean read lengths of 150 bp (Table S2). Six carbapenem-resistance genes were identified, with *bla*_NDM-1_ being the most prevalent (75%, 48/64), followed by *bla*_IMP-4_ (7.8%, 5/64) and *bla*_KPC-2_ (6.2%, 4/64). Sequence typing revealed 21 STs, with ST14 being the predominant (46.9%; 30/64), followed by ST433 (10.9%, 7/64) (Fig. 1a; Table S3). Only three isolates (4.7%) were from ST11.

Virulence gene analysis revealed that 22 isolates (34.4%) harbored *ybt* within integrative conjugative element in *K. pneumoniae* (ICEKp) structures: 12 isolates carried *ybt*16 (ICEKp12), five carried *ybt*9 (ICEKp3), four carried *ybt*10 (ICEKp4), and one carried *ybt*17 (ICEKp10). The colibactin gene cluster (*clb*) was detected in a single isolate, also associated with an ICE structure. Critically, no isolates harbored other hypervirulence genes (*rmpA*, *rmpA2*, *iuc*, *iro*) typically associated with virulence plasmids. This pattern corroborates our previous findings that CRKP isolates in pediatric populations exhibit reduced virulence potential relative to adult strains (23).

Phylogenetic analysis based on core-genome single-nucleotide polymorphisms (cgSNPs) delineated distinct lineages for the STs (Fig. 1b). Most isolates within each ST exhibited high genetic similarities, with a mean cgSNP difference of 8 (0–29), except for ST14 (mean cgSNPs: 128), which displayed greater divergence. ST14 could be subdivided into two clones, ST14-C1 and ST14-C2, with intra-clonal diversities of only 6 (0–18) cgSNPs and a difference of 251 cgSNPs between them. The clones also differed in AMR genes, with *tet*(A) exclusively in ST14-C1 and *qnrS1* and trimethoprim-resistance genes primarily in ST14-C2 (Fig. 1b; Fig. S1).

Phylogenetic analysis of ST14 and ST433 strains together with publicly available genomes revealed their distinct evolutionary origins (Fig. S2). ST14-C1 clustered with Southeast Asian isolates, while ST14-C2 showed closest affinity to Canadian strains, supporting North American ancestry and indicating multiple independent introductions from geographically distinct regions (Fig. S2a) (23). Finally, ST433 isolates clustered tightly with strains from Hunan Province, suggesting domestic transmission or shared ancestral sources (Fig. S2b).

### Characterization of the plasmids encoding carbapenemase

**Fig 2:**
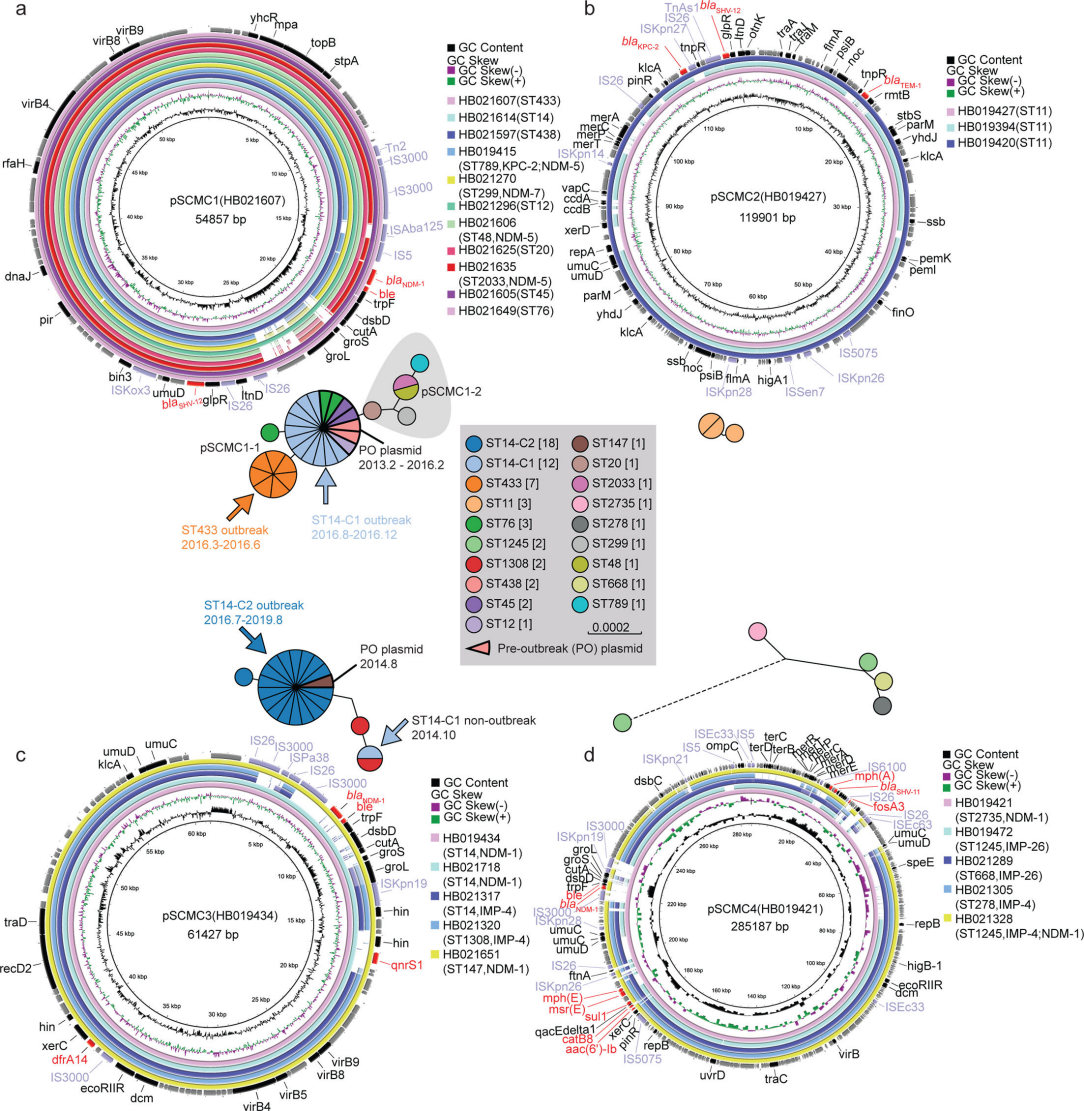
Characterization of the carbapenemase-encoding plasmids. (**a–d**) Circular plots of the four plasmid types from various isolates. Gene symbols were labeled in the outermost circle, with the ARGs in red and the insertion sequences in purple. Insets: the maximum-likelihood phylogenies of the four plasmid types. Nodes were color-coded by the bacterial STs as in the key.

We predicted four primary carbapenemase-carrying plasmids (pSCMC1-4) in the CRKP isolates (23) and selected seven representatives, encompassing all of pSCMC1-4, for Nanopore sequencing. No plasmids related to virulence genes were identified. Seven hybrid assemblies generated from Illumina and Nanopore sequencing data had an estimated average depth of coverage of approximately 190× (Table S2). Complete chromosomes were obtained for all seven isolates, plus 21 circulated plasmids, including pSCMC1-4. The *bla*_NDM-1_-encoding pSCMC1 plasmid was identified in ST433 strains, all but one ST14-C1 strain, and 12 strains from seven other STs (Fig. 2a). Phylogeny (Fig. 2) divided pSCMC1 (IncX3) into two subclades: pSCMC1-1, primarily in NICUs and preterm neonates in the neonatal units, and pSCMC1-2, in full-term neonates in the neonatal unit. The pSCMC3 (IncN) plasmid, carrying *bla*_NDM-1_ or *bla*_IMP-4_ genes, was found in ST14-C2 strains, the earliest ST14-C1 isolate (HB021317) from 2014, and three strains from two other STs (Fig. 2c). Furthermore, the *bla*_KPC-2_-carrying pSCMC2 (IncFII) was exclusively in ST11 strains, and the *bla*_IMP_- or *bla*_NDM-1_-carrying pSCMC4 (IncU) was in five other strains (Fig. 2b and d).

To further investigate the mobility and structural context of carbapenemase genes, we analyzed the genetic environments surrounding *bla*_NDM-1_ and *bla*_KPC-2_ in the seven isolates subjected to Nanopore sequencing. The complete plasmid sequences enabled us to resolve the local genetic structures of these resistance genes. We found that *bla*_KPC-2_ was carried on the pSCMC2 plasmid within the ISKpn27-*bla*_KPC-2_-IS26 conserved structure (Fig. S3a). In contrast, *bla*_NDM-1_ was found in three distinct genetic contexts across different plasmid types. The pSCMC1 plasmid carried *bla*_NDM-1_ within the IS3000-ISAba125-IS5-*bla*_NDM-1_-*ble-trpF-dsbD-cutA-groS-groL*-IS26 structure. The pSCMC3 plasmid carried *bla*_NDM-1_ within the IS3000-*bla*_NDM-1_-*ble-trpF-dsbD-cutA-groS-groL*-ISKpn19 structure. The pSCMC4 plasmid carried *bla*_NDM-1_ within the IS3000-*bla*_NDM-1_-*ble-trpF-dsbD-cutA-groS-groL*-ISKpn19-IS3000 structure (Fig. S3b). These findings demonstrate a strong correlation between the carbapenemase gene environment and the associated plasmid type.

### Assessing risk factors associated with CRKP persistence

We identified key risk factors facilitating the persistence and dissemination of CRKP isolates by examining those shared by isolates within specific time frames. For instance, the rapid spread of a cluster of six ST14-C2 isolates in June 2019 suggested that bacterial genotypes, including STs and sub-ST clones, are significant risk factors.

**Fig 3:**
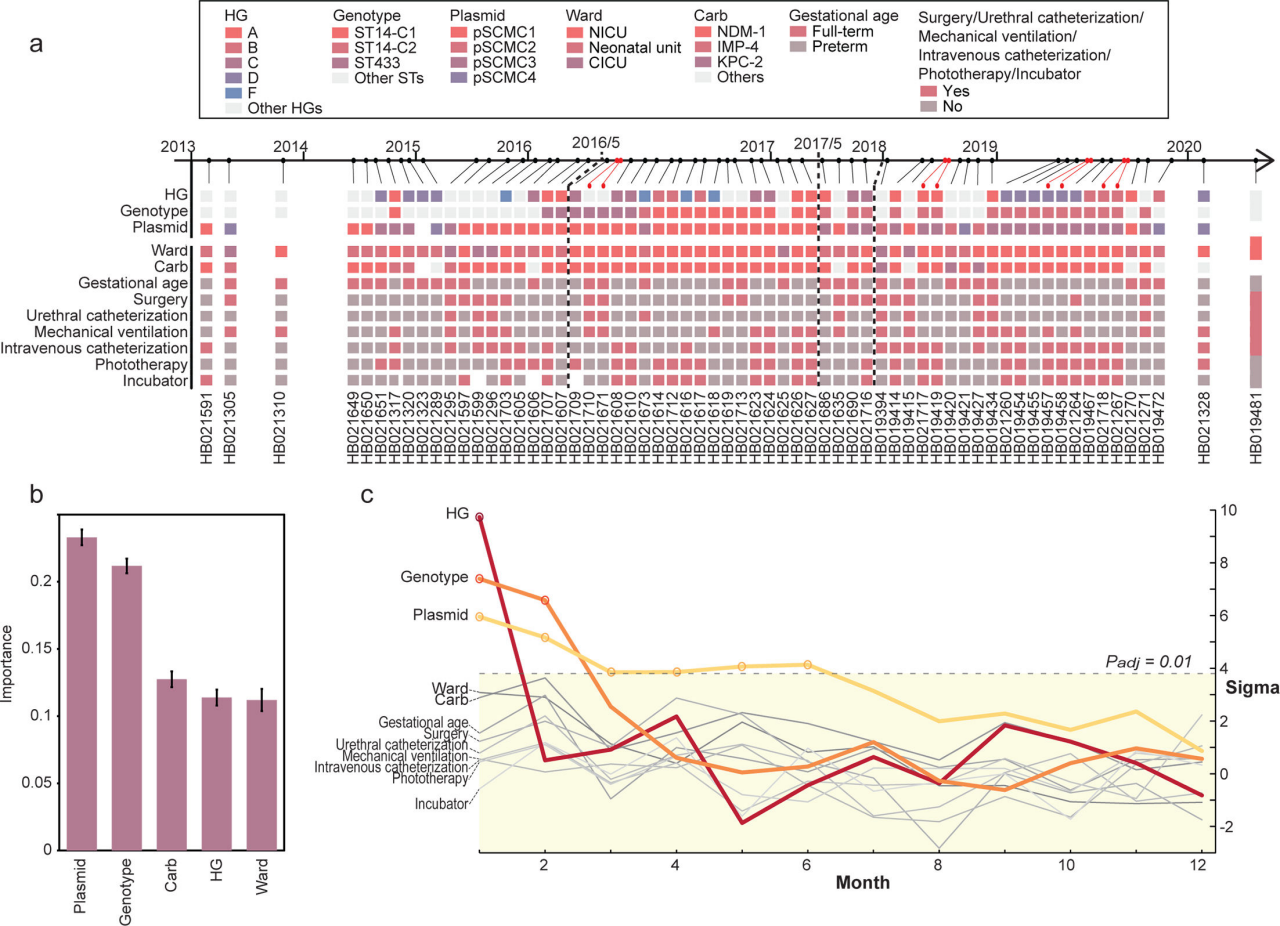
The assessment of the risk factors contributing to the dissemination of CRKP isolates over time. (**a**) Timeline of 64 strains isolated. Relevant clinical and genomic information was presented in the heatmap below. Strains isolated on the same day were marked with red circles. Dashed lines indicated the time points of plasmid shifts and the exclusive entry of plasmids into the NICU. Carb, carbapenem-resistance genes. (**b**) The permutation importance of most contributing features in random forest regression model. Only features with >0.1 contributions were visualized. Error bars indicate standard variations in permutations. (**c**) The significance of frequencies (*y*-axis) in obtaining isolates with the same clinical metadata (i.e., HG or ward) or bacterial features (i.e., genotype or plasmid) in a certain period of time (*x*-axis). The significance was calculated by comparing to 1,000 permutations and measured by sigma, numbers of folds of deviation to the normal distributions estimated by the permuted data sets. The colored circles show significant values (*P* < 0.01) after the Bonferroni adjustments, and their associated lines were also color-coded. The actual and permuted frequencies of the three significant factors (HG, genotype, and plasmid) were also shown in Fig. S1.

To rigorously test the importance of these potential factors, a novel analytical framework that accounts for temporal differences between isolates was developed. We compared 64 CRKP isolates by their isolation time intervals, clinical characteristics, genotypes, plasmid content, and antimicrobial resistance genes (ARGs) (Fig. 3a). Considering the strong correlation between plasmid types and the genetic environment of carbapenemase genes, we included only plasmid content in the risk factors analysis to avoid redundancy. The random forest regression model, trained on 2,080 pairwise comparisons, achieved a mean squared error of 0.86 during fivefold cross-validation. These metrics demonstrated the model’s strong predictive power in identifying time intervals based on genomic and clinical data. The factors contributing most to CRKP persistence were carbapenemase-carrying plasmids (0.233 ± 0.006), followed by bacterial genotypes (0.212 ± 0.005), carbapenemase genes (0.127 ± 0.006), HGs (0.114 ± 0.006), and hospital wards (0.112 ± 0.008) (Fig. 3b). This highlights the role of plasmid-mediated transmission in the long-term persistence of CRKP across neonatal units.

To further explore the time scales on which these factors influenced CRKP transmission, we employed a permutation test. The test identified the same set of five top factors, with three showing statistical significance: HGs, bacterial genotypes, and plasmids (Fig. 3c; Fig. S4). These factors exerted their effects over distinct time scales. Patients in the same HGs shared significantly more CRKP infections within 1 month, with the influence of HG membership diminishing after 2 months, indicating its role as a short-term mediator. In contrast, bacterial genotypes and plasmids contributed to transmissions over 1–2 months and 1–6 months, respectively, marking genotypes as mid-term mediators and plasmids as long-term mediators. Notably, plasmids had a more prolonged effect on transmission than bacterial genotypes, emphasizing their role in the long-term persistence of CRKP across multiple sequence types.

### Risk factor 1: bacterial genotypes for NICU outbreaks

Bacterial genotypes are key mediators for HAI outbreaks, typically associated with the spread of single clones (24, 25). We identified three major clonal outbreaks in the NICU, each involving ≥5 isolates, attributed to ST14 or ST433.

**Fig 4:**
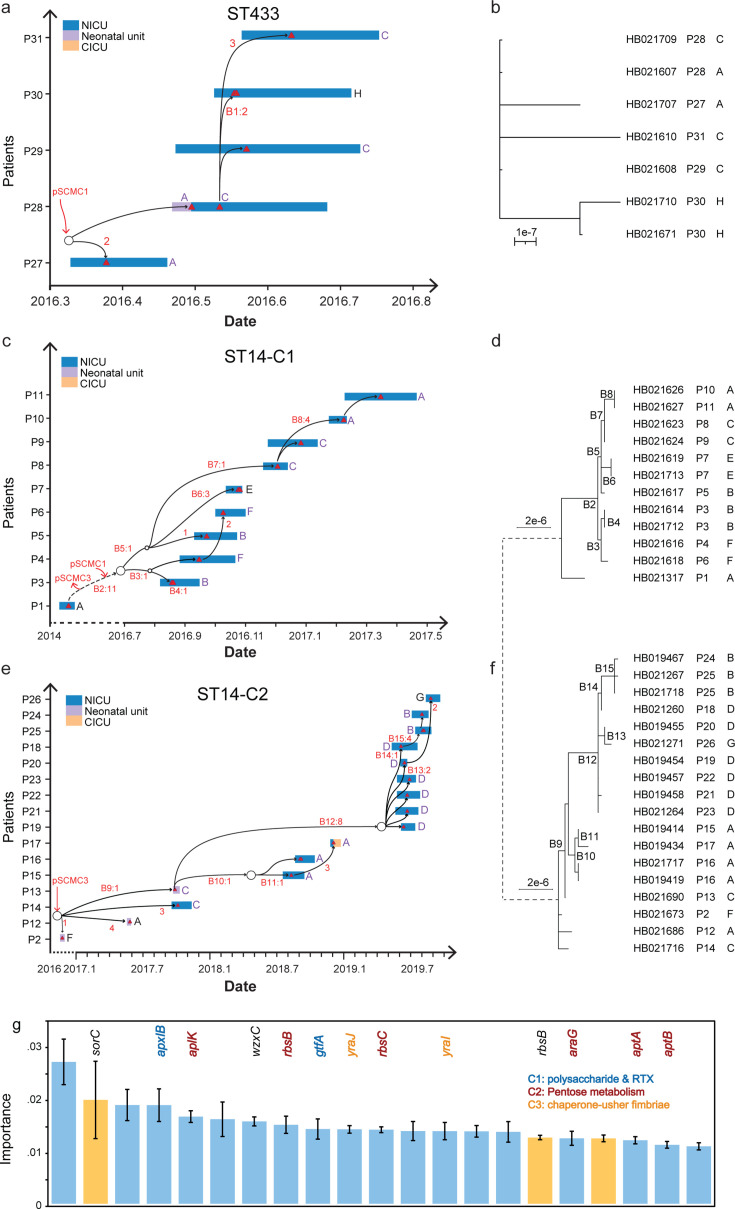
Clonal spread pathways of the ST14 and ST433 CRKPs in the NICU. (**a, c, e**) The reconstructed spread pathways of ST433, ST14-C1, and ST14-C2 according to the core genomic phylogenies (**b, d, f**). Rectangles indicate the period from patient admission to discharge, and colors indicate the ward of admission. Triangles indicate the isolation time of isolates. Red fonts indicate the corresponding branches and the number of cgSNPs. Circles represent potential intermediate transmitters that have not been sampled. (**b, d, f**) The maximum-likelihood phylogeny of ST433, ST14-C1, and ST14-C2. (**g**) The permutation importance of most contributing features in random forest classification model of outbreak-associated genotypes. Only features with >0.01 contributions were visualized. Error bars indicate standard variations in permutations. Genes in the three gene clusters were color-coded as in the key.

The first outbreak, caused by ST433, occurred between March and July 2016, affecting four patients (Fig. 4a and b). Patients P27 and 28, both handled by HG-A, were the initial cases. P28 was later transferred to HG-C in the NICU and likely became a super-spreader, infecting P29 and P31, both also handled by HG-C, and P30, handled by HG-H.

The second outbreak was associated with ST14-C1 (Fig. 4c and d). The first ST14-C1 isolate in 2014 was not directly linked to the outbreak, differing by 11 cgSNPs and a plasmid, and demonstrated the long-term persistence of ST14-C1. From August to October 2016, a cluster of four patients was identified. P4 and P6, handled by HG-F, formed a tight phylogenetic cluster, indicating direct transmission. After a brief hiatus, the outbreak resumed, with four more patients infected between December 2016 and March 2017. Direct transmissions were again observed, with P8 and P9, both handled by HG-C, and P10 and P11, handled by HG-A.

The third and largest transmission chain, involving ST14-C2, spanned 2 years and caused intersecting outbreaks (Fig. 4e and f). The first two ST14-C2 isolates were likely sporadic infections in the neonatal unit. Subsequently, the pathogen infected three patients handled by HG-C between September and October 2017, after which it remained undetected for a time. The second wave involved three patients handled by HG-A from August to December 2018. The most substantial outbreak followed in June 2019, involving nine patients. This outbreak began with six patients handled by HG-D and later spread to P25 managed by HG-B and P26 by HG-G. Notably, the isolate from P25 established secondary transmission to P24, also handled by HG-B.

To further investigate the genetic differences between outbreak-associated and sporadic genotypes, the accessory genome was analyzed. We constructed a pan-genome of 11,226 genes for the isolates and established a random forest classification model based on 7,371 accessory genes. The model predicted outbreak-associated genotypes with 100% accuracy, indicating strong performance in identifying this genotype based on accessory gene presence.

Twenty-one genes contributed significantly to the classification, with at least a 0.01 impact score (Fig. 4g). These included 13 genes from three gene clusters: one cluster containing the *gtfA* and *apxIB* genes, encoding UDP-GlcNAc-peptide N-acetylglucosaminyltransferase and RTX-I translocation ATP-binding protein, respectively; a second cluster of eight genes involved in pentose transport and metabolism; and a third cluster of three genes encoding chaperone-usher fimbriae. These genes were present in almost all outbreak-associated genotypes but were rarely found in sporadic isolates.

### Risk factor 2: healthcare groups as mediators of transmissions

HGs played significant roles in the transmission of CRKPs during NICU outbreaks. As noted, 81% (25/31) of infection clusters during outbreaks included multiple patients from the same HG (Fig. 4), with nearly all HG-mediated transmissions persisting for only 1 month. This pattern demonstrated that HGs acted as short-term mediators of transmission. Interestingly, none of the minor STs were associated with HG-mediated transmissions, indicating that this mechanism is more prominent in major outbreaks. The three HGs most frequently involved in transmission were HG-C (7 out of 8 patients), HG-A (7 out of 10 patients), and HG-D (6 out of 11 patients).

Inter-HG transmissions were also observed, particularly between HG-A and HG-C, likely driven by patient transfers. For example, patient P28, initially managed by HG-A, was transferred to HG-C, where further transmission occurred. These findings highlight the role of HG dynamics, particularly patient movement, in facilitating the spread of CRKP within the NICU.

### Risk factor 3: plasmids for long-term persistence

**Fig 5:**
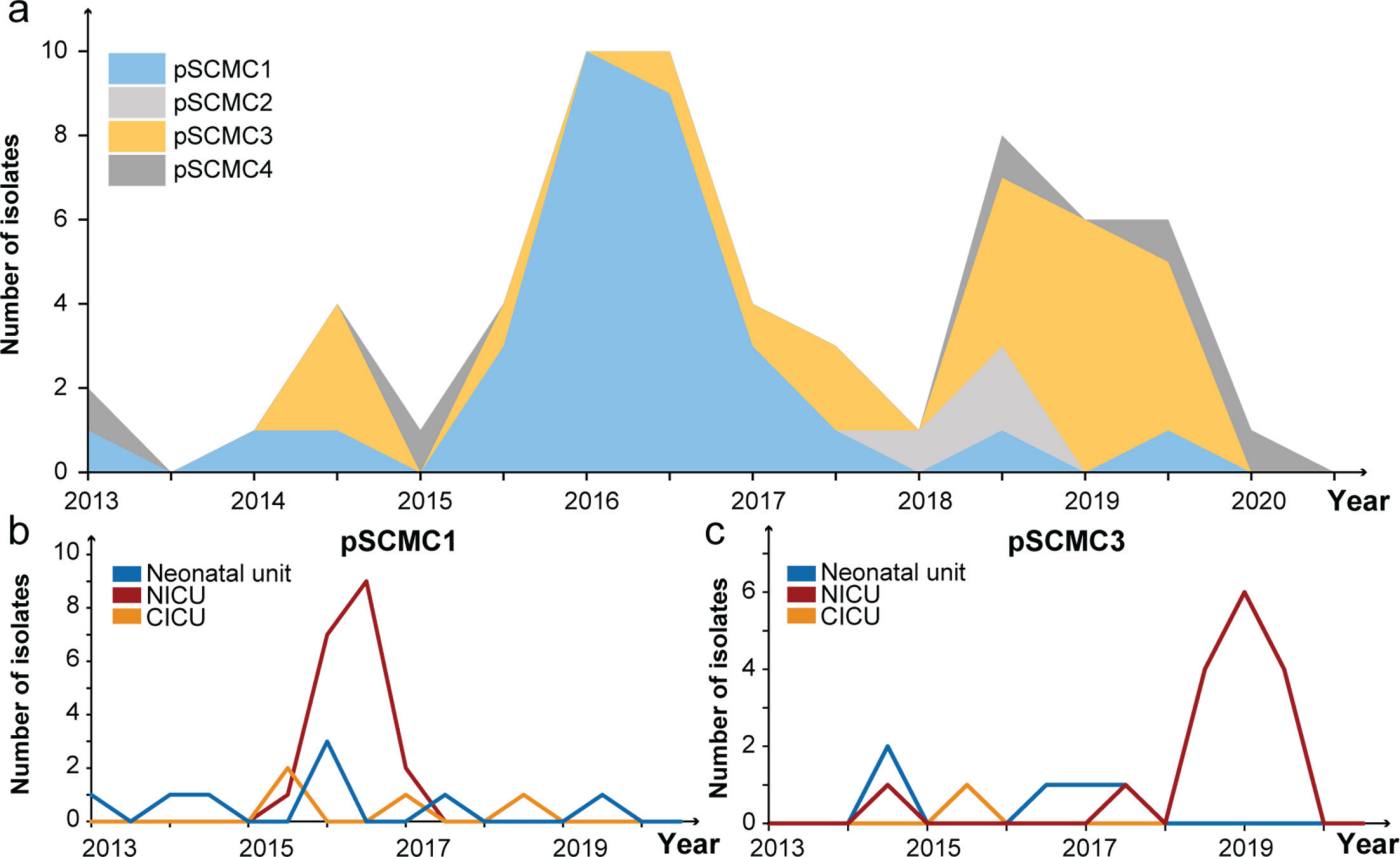
The temporal dynamics in the prevalence of carbapenemase-encoding plasmids. (**a**) Numbers of isolates each year, color-coded by their associated carbapenemase-encoding plasmids. (**b**) Numbers of neonates infected by pSCMC1-carrying isolates in different wards each year. (**c**) Numbers of neonates infected by pSCMC3-carrying isolates in different wards each year.

Our analysis indicated that plasmids played a crucial role in the year-long persistence of CRKP isolates. Specifically, 19 of the 21 NICU isolates collected before May 2017, scattered across four distinct STs, were associated with pSCMC1-1. In contrast, 16 of the 18 isolates collected afterward were associated with pSCMC3 (Fig. 5a). Notably, pSCMC1-1 shifted from being primarily outside the NICU (7/10 isolates) before May 2016 to exclusively within the NICU (16/16) afterward, resulting in consecutive disease outbreaks of ST433 and ST14-C1 in 2016 (Fig. 5b). A similar pattern was observed with pSCMC3, which transitioned from being predominantly found in the neonatal unit (5/8) to exclusively present in the NICU (14/14) after 2018, leading to the ST14-C2 outbreak in 2019 (Fig. 5c).

Importantly, the persistence of these plasmids was not confined to specific STs. The pSCMC1-1 plasmid was carried by 11 distinct STs, while pSCMC3 was found in three different STs (Fig. 1). This study highlights for the first time that plasmids, specifically pSCMC1 and pSCMC3, play a critical role in the long-term persistence and transmission of CRKP across multiple bacterial genotypes. Unlike clonal dissemination, plasmid-mediated transmission enables CRKP to persist in the NICU environment even after clonal strains subside, underscoring plasmids as key long-term mediators of outbreaks.

**Fig 6:**
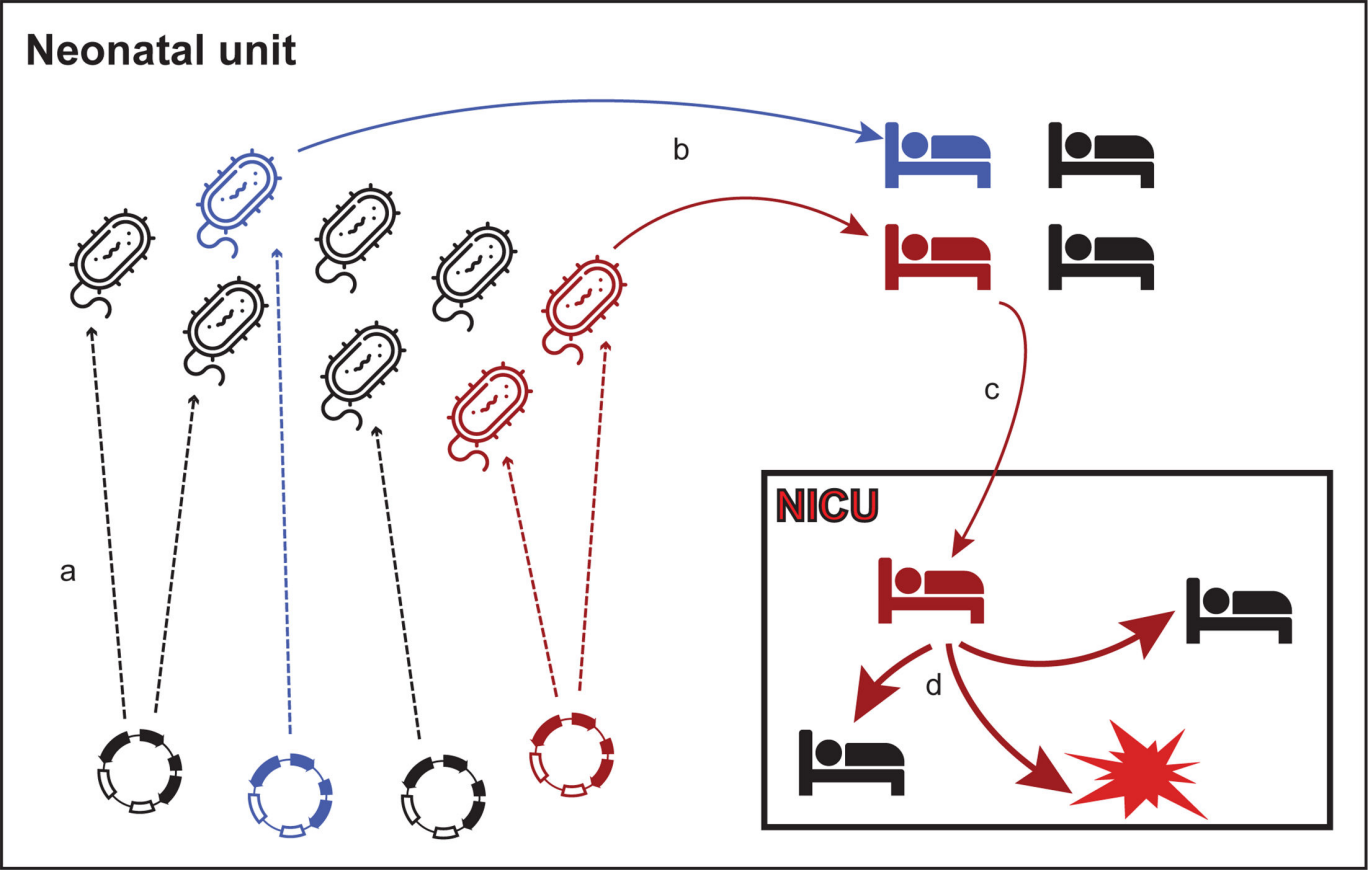
Overview of the transmission trajectory from the neonatal unit to the NICU. (**a**) The *Klebsiella pneumoniae* isolates with diverse STs acquired carbapenemase-encoding plasmids. (**b**) Patients admitted to the neonatal unit were infected with CRKP isolates. (**c**) Some infected patients were transferred to the NICU. (**d**) Rapid transmission of CRKP isolates in the NICU, resulting in disease outbreaks.

From a plasmid-centric perspective, the NICU outbreaks shared notable characteristics. Pre-outbreak (PO) plasmids, genetically indistinguishable from those found during outbreaks, were detected in the neonatal unit 2–3 years before the outbreaks. These plasmids were initially carried by *Klebsiella* isolates with diverse STs. Some of these PO plasmids were inadvertently introduced into the NICU, possibly through patients, such as the case of ST433 in patient P28, leading to recurring transmissions and subsequent outbreaks (Fig. 6). Monitoring these PO plasmids in both the neonatal and NICU units could serve as an early warning system for impending outbreaks, offering a crucial opportunity for timely intervention and containment efforts.

## DISCUSSION

This study provides a comprehensive analysis of CRKP transmission dynamics in neonatal units over 8 years, identifying bacterial genotypes, HGs, and plasmids as the key drivers of CRKP persistence and outbreaks. By combining genomic, clinical, and epidemiological data, we have identified the factors driving CRKP outbreaks and provided valuable insights into infection control strategies in critical care environments.

Our use of random forest models successfully identified critical factors contributing to the persistence and spread of CRKP. The model explained 86% of the variance in isolation times, confirming its robustness. Our analyses aligned with previous models (26, 27) for the role of bacterial clonal expansion and healthcare groups in the dissemination of CRKPs and further highlighted the previously overlooked factors, particularly plasmids, as the most significant factor for long-term persistence, underscoring the need for surveillance that targets both plasmid dynamics and clonal spread in NICU settings.

The temporal differences in the influence of these factors were confirmed by permutation tests. Healthcare groups were identified as key mediators of short-term transmission, with their impact diminishing after 1 month. In contrast, bacterial genotypes and plasmids contributed to transmission over longer periods, with plasmids playing a more prolonged role in CRKP outbreaks, persisting up to 6 months. This underscores the importance of monitoring both short-term interactions within healthcare groups and long-term plasmid-based transmission pathways.

The clonal spread of specific genotypes, particularly ST14 and ST433, was a primary driver of NICU outbreaks. ST14 is one of the most abundant STs internationally, associating with both *bla*_NDM_ and *bla*_OXA_ (23). Our early analyses showed that the two clones, ST14-C1 and ST14-C2, represent independent international transmissions into China (23), complicating outbreak dynamics (Fig. 4). Furthermore, none of the neonatal CRKPs encode major virulence genes, i.e., *rmpA/rmpA2* or *iuc* (17), and ST11, accounting for over 70% of HAIs in adults (28), was isolated only three times, highlighting the distinct genetic context of the CRKPs infecting neonates and adults. Our previous investigation revealed that 40% of ST11 isolates originated from patients with recent surgical interventions at external hospitals, indicating ST11 represents primarily imported strains lacking sustained transmission capacity in pediatric settings (23). The distinct immunological and microbiological environments of neonates may render ST11 less capable of establishing persistent colonization compared to the adapted ST14 and ST433 lineages. Our analysis highlighted the rapid spreading of pathogenic clones in the NICU (Fig. 4), each with distinct temporal patterns and transmission dynamics, suggesting that specific bacterial strains can persist in the hospital environment over time (29).

Our analysis highlights the critical role of HGs in short-term CRKP transmission (Fig. 3). Over 80% of infection clusters involved patients managed by the same HGs, with transmission events predominantly occurring within 1 month (Fig. 4). Intra-group interactions, including shared medical staff and equipment, play a significant role in pathogen spread (8, 30), underscoring the importance of stringent infection control measures and targeted interventions within these units. The transmission between HGs, particularly between HG-A and HG-C in the ST433 outbreak, underscores the role of patient movement in amplifying the transmission network, consistent with previous studies where pathogens were disseminated between hospitals during patient transfers (28). This necessitates the implementation of robust patient transfer protocols to mitigate cross-group transmission risks (31, 32).

The ST14-C1 and ST14-C2 outbreaks presented a more complex transmission pattern, characterized by periods of apparent dormancy followed by resurgence. These suggest the existence of underlying reservoirs within the hospital environment or intermittent reintroduction from external sources (33). High bacterial loads of *K. pneumoniae* have been reported on various surfaces and medical equipment in ICUs (8), including incubators, suction tips, and maternity beds. This environmental persistence underscores the need for rigorous cleaning protocols and continuous surveillance of hospital environments (34). Preventing CRKP from lingering on surfaces or in equipment could help prevent future outbreaks.

The dissemination of highly similar IncX3 *bla*_NDM-5_-carrying plasmids among multiclonal *K. pneumoniae* strains in children has been described, with evidence indicating that the conserved type IV secretion system plays a key role in promoting plasmid stability and persistence (35). Additionally, a plasmid-mediated outbreak of *bla*_NDM-1_-producing *K. pneumoniae* ST105 was reported among neonates in Yunnan, further highlighting the critical role of plasmids in the clinical spread of carbapenem resistance (9). In line with these findings, our findings highlight the importance of plasmid surveillance in preventing long-term CRKP persistence. Unlike clonal spread, which tends to drive acute outbreaks, plasmids enable horizontal gene transfer across different strains, promoting the long-term survival of CRKP in hospital settings. The temporal shift in the prevalence of plasmids pSCMC1-1 and pSCMC3 correlated with the epidemiology of CRKP infections (Fig. 5). The widespread distribution of the plasmids across diverse *Klebsiella* isolates (Fig. 2) highlights their roles in facilitating horizontal gene transfer and sustaining CRKP reservoirs within the hospital environment (36, 37). PO plasmids, detected years before outbreaks, were genetically indistinguishable from outbreak plasmids, emphasizing their potential role as early indicators of impending outbreaks (Fig. 6). Surveillance of these plasmids in both neonatal and NICU units could allow for timely interventions to prevent future outbreaks.

Accessory genes associated with CRKP outbreaks further reveal the mechanisms behind bacterial persistence (Fig. 5d). The first cluster includes *gtfA* and *apxIB*, involved in surface polysaccharide synthesis and RTX toxin secretion (38, 39), respectively, both of which enhance bacterial adhesion, biofilm formation, and immune evasion (40, 41). The second cluster relates to pentose transport and metabolism, providing a survival advantage in nutrient-limited environments (42). The third cluster encodes chaperone-usher fimbriae, promoting bacterial attachment to host tissues (43). The presence of these genes in outbreak-associated isolates highlights their potential role in enhancing CRKP’s ability to persist in the NICU environment, evade host immune responses, and spread between patients.

Our findings have important implications for infection control in NICUs. The frequent intra- and inter-group transmissions call for enhanced infection control measures, particularly around patient transfers and within high-risk HGs (44, 45). Additionally, the role of plasmids in sustaining long-term persistence necessitates a dual focus on both clonal and plasmid surveillance to manage and prevent CRKP outbreaks (Fig. 6). This discovery shifts the focus of infection control from solely targeting clonal dissemination to also encompassing plasmid surveillance and intervention. The novel identification of plasmid-mediated persistence reveals potential early warning signs of outbreaks, offering a new dimension for preventing recurrent CRKP transmission.

We acknowledge the limitations of our study. This study focused exclusively on CRKP isolates from neonates, which may have limited our ability to fully capture the broader transmission dynamics within the hospital environment. In addition, we were unable to retrospectively sample NICU environments or the carbapenem-susceptible isolates, which likely served as important reservoirs for the plasmids. Future multi-center studies combining environmental surveillance and genomic data could provide deeper insights into CRKP reservoirs and transmission pathways. Another limitation was the relatively small number of isolates, which may affect the robustness and generalizability of the machine learning-based analysis. To mitigate this, we applied fivefold cross-validation and performed hyperparameter tuning to reduce overfitting and improve model stability, as described in the Materials and Methods section. Nevertheless, we acknowledge that the findings should be interpreted with caution and viewed as preliminary. Future studies involving larger and more diverse data sets will be essential to validate and strengthen the conclusions drawn from this work.

In conclusion, this study sheds light on the complex transmission dynamics of CRKP in neonatal care settings. By combining genomic, clinical, and epidemiological data, we identified the critical roles of clonal spread, healthcare group dynamics, and plasmid-mediated persistence. Addressing these factors through comprehensive surveillance and targeted interventions is essential to mitigating CRKP outbreaks in NICUs. This research not only advances our understanding of CRKP transmission but also lays the groundwork for more effective strategies to combat the spread of multidrug-resistant pathogens in hospital environments.

## Data Availability

The raw sequence data reported here have been deposited in the Genome Sequence Archive (46) in National Genomics Data Center (47), China National Center for Bioinformation/Beijing Institute of Genomics, Chinese Academic of Science (PRJCA012323, GSA: CRA008499; CRA016970), and are publicly accessible (https://ngdc.cncb.ac.cn/bioproject/browse/PRJCA012323). The entire set of ST14 and ST433 public genomes is available in EnteroBase and GenBank, with the accession codes in Tables S1 and S4.

## SUPPLEMENTAL MATERIAL

Fig. S1 msystems.00909-25-s0001.eps The recombination-removed phylogeny of ST14 *K. pneumoniae* isolates.

Fig. S2 msystems.00909-25-s0002.eps Global phylogenetic analysis of ST14 and ST433 genomes.

Fig. S3 msystems.00909-25-s0003.eps Genetic environments of *blaKPC-2* and *blaNDM-1* genes identified in representative CRKP isolates based on Nanopore sequencing.

Fig. S4 msystems.00909-25-s0004.eps Measuring the proportions of isolates sharing the healthcare group, genotype, and plasmid in a certain period.

Supplemental tables msystems.00909-25-s0005.xlsx Tables S1 to S4.

ASM does not own the copyrights to Supplemental Material that may be linked to, or accessed through, an article. The authors have granted ASM a non-exclusive, world-wide license to publish the Supplemental Material files. Please contact the corresponding author directly for reuse.
